# Genome-Wide Association Study and QTL Meta-Analysis Identified Novel Genomic Loci Controlling Potassium Use Efficiency and Agronomic Traits in Bread Wheat

**DOI:** 10.3389/fpls.2020.00070

**Published:** 2020-02-18

**Authors:** Luqman Bin Safdar, Tayyaba Andleeb, Sadia Latif, Muhammad Jawad Umer, Minqiang Tang, Xiang Li, Shengyi Liu, Umar Masood Quraishi

**Affiliations:** ^1^ Key Laboratory of Biology and Genetics Improvement of Oil Crops, Oil Crops Research Institute of Chinese Academy of Agricultural Sciences, Ministry of Agriculture and Rural Affairs, Wuhan, China; ^2^ Department of Plant Sciences, Quaid-i-Azam University, Islamabad, Pakistan; ^3^ Zhengzhou Fruit Research Institute, Chinese Academy of Agricultural Sciences, Zhengzhou, China

**Keywords:** potassium use efficiency, single-locus GWAS, multi-locus GWAS, marker-trait associations, meta-QTL

## Abstract

Potassium use efficiency, a complex trait, directly impacts the yield potential of crop plants. Low potassium efficiency leads to a high use of fertilizers, which is not only farmer unfriendly but also deteriorates the environment. Genome-wide association studies (GWAS) are widely used to dissect complex traits. However, most studies use single-locus one-dimensional GWAS models which do not provide true information about complex traits that are controlled by multiple loci. Here, both single-locus GWAS (MLM) and multi-locus GWAS (pLARmEB, FASTmrMLM, mrMLM, FASTmrEMMA) models were used with genotyping from 90 K Infinium SNP array and phenotype derived from four normal and potassium-stress environments, which identified 534 significant marker-trait associations (MTA) for agronomic and potassium related traits: pLARmEB = 279, FASTmrMLM = 213, mrMLM = 35, MLM = 6, FASTmrEMMA = 1. Further screening of these MTA led to the detection of eleven stable loci: *q1A, q1D, q2B-1, q2B-2, q2D, q4D, q5B-1, q5B-2, q5B-3, q6D,* and *q7A*. Moreover, Meta-QTL (MQTL) analysis of four independent QTL studies for potassium deficiency in bread wheat located 16 MQTL on 13 chromosomes. One locus identified in this study (*q5B-1*) colocalized with an MQTL (*MQTL__11_*), while the other ten loci were novel associations. Gene ontology of these loci identified 20 putative candidate genes encoding functional proteins involved in key pathways related to stress tolerance, sugar metabolism, and nutrient transport. These findings provide potential targets for breeding potassium stress resistant wheat cultivars and advocate the advantages of multi-locus GWAS models for studying complex traits.

## Introduction

Potassium (K) plays a critical role in plant growth and development, elucidated by its multidimensional capacity of regulating the plant physiological systems such as enzymes activation, membrane potential, osmoregulation, photosynthesis, and osmotic balance (Clarkson and Hanson, 1980; Pettigrew, 2008; Wang and Wu, 2017). However, K-deficiency implicates restrictions in sustainable plant growth and development (Rengel and Damon, 2008; Fageria and Moreira, 2011; Wang and Wu, 2015). To fulfil the nutrient demands of food crops and to achieve a higher grain yield, fertilizer applications are widely used (Fageria et al., 2009; Fageria and Moreira, 2011; Lotter, 2015). But the high application of fertilizers is also a serious challenge to not only the environment but also the economy of common farmers (Lægreid et al., 1999), for instance, higher nutrient uptake leads to: (a) nutrient mining in low input systems which results in leaching to water bodies, (b) higher fertilizer demand in high input cropping systems which is farmer unfriendly. A more affordable and sustainable approach adapted by plant breeders to reduce the excessive fertilizer use is the development of cultivars with high nutrient efficiency (Baligar et al., 2001; Trehan, 2005; Hawkesford et al., 2016; Sarkar and Baishya, 2017). As for K, different plant species or even the different genotypes of the same species are known to have a varied K-uptake and utilization efficiency (Pettersson and Jensen, 1983; Guoping et al., 1999). This provides a possibility for the genetic dissection of crop KUE (Wang and Wu, 2015). In the past, K-acquisition by roots (K-uptake efficiency [KUpE]) was considered to be of primary importance by the scientists. However, in calcareous agricultural soils, nutrient uptake by plants is extremely limited (Fageria and Nascente, 2014) due to a high nutrient fixation rate in the soil (Friesen et al., 1997). Therefore, K-use efficiency (KUE) cannot be increased by KUpE alone (Gamuyao et al., 2012), and thus the K-utilization capacity of plants (KUtE) must also be improved to achieve a higher KUE (Rose and Wissuwa, 2012). Plants with high KUE have a higher KUpE and KUtE (El Bassam, 1997; Zhang et al., 2007), thus KUE is defined as a product of the two (Sandaña, 2016). Several studies in the past have reported the genotypic variation of KUE in wheat (Guoping et al., 1999; George et al., 2002; Damon and Rengel, 2007; Pettigrew, 2008; Rengel and Damon, 2008; Wang and Wu, 2015). This suggests that the genetic improvement of crops can be carried out by selecting important quantitative trait loci (QTL) associated to KUE. However, despite the importance of genetic dissection of this complex trait, only a few QTL studies have been reported in wheat using bi-parental mapping populations (Guo et al., 2012; Kong et al., 2013; Zhao et al., 2014; Gong et al., 2015). Genetic maps used in these various studies can be integrated at one place to identify consensus genomic region called Meta-QTL (MQTL), independent of population type and genotype/environment interaction (Quraishi et al., 2017). This approach of identifying MQTL by meta-analysis was first proposed by Goffinet and Gerber (2000), and has since been applied in many crops including wheat (Griffiths et al., 2009; Gegas et al., 2010; Quraishi et al., 2017).

Recent advances in molecular biology and next generation sequencing along with the discovery of new genome analysis tools have helped identifying high-throughput single nucleotide polymorphisms (SNPs) that are utilized to construct high resolution genetic maps for genome-wide association study (GWAS) (Hyten et al., 2010; Song et al., 2016; Su et al., 2016; Wu et al., 2016). GWAS is considered as a significant approach to study genetic variants in a large population as it saves time and cost of developing a bi-parental population, deduces multi-allelic variations to help identify the most favorable alleles of a target trait in a single analysis, and it is more powerful and easy to fine map QTL due to a higher resolution resulting from a high genetic diversity (Breseghello and Sorrells, 2006; Atwell et al., 2010). GWAS takes complete advantage of all the recombination events occurring in the evolution of a natural population (Chang et al., 2018). GWAS has been used to understand the genetic basis of complex traits in various plant and animal species (Hirschhorn and Daly, 2005; McCarthy et al., 2008; Ingvarsson and Street, 2011; Bush and Moore, 2012). Conventionally used single-locus model for GWAS is the mixed linear model (MLM) approach, the so-called Q+K model, that uses the population structure (Q) and kinship matrix (K) (Yu et al., 2006). Since the publication of MLM, many different MLM models have been reported (Kang et al., 2008; Zhang et al., 2010; Zhou and Stephens, 2012; Zhou et al., 2013). MLM models conduct one-dimensional genome scanning to test one marker at a time, these models can handle a large proportion of markers, e.g., up to a million markers (Wang et al., 2016). However, most complex traits such as nutrient use efficiency are usually controlled by multiple loci, and thus MLM based models are never accurate to estimate the marker effects for such traits. Another problem with MLM based models is that the stringent criterion of significance for marker selection such as Bonferroni correction does not allow many significant markers to be detected (Wang et al., 2016; Chang et al., 2018). Multi-locus mixed linear models have been developed to address this problem because they can be used to detect powerful marker-trait associations (MTA) using lower significance criterion as no Bonferroni correction is applied (Wang et al., 2016; Chang et al., 2018; Lü et al., 2018; Ma et al., 2018; Peng et al., 2018; Zhang et al., 2018). Ever since the first multi-locus random-SNP-effect mixed linear model (mrMLM) was proposed by Wang et al. (2016), a series of models has been published in various studies, e.g., pLARmEB (Zhang et al., 2017), FASTmrMLM (Zhang and Tamba, 2018), and FASTmrEMMA (Wen et al., 2018).

In this study, one single-locus GWAS (SL-GWAS) model and four multi-locus GWAS (ML-GWAS) models were used to identify significant MTA for KUE and agronomic traits in a historical bread wheat diversity panel of Pakistan. Furthermore, a meta-analysis of all the reported QTL studies (related to K-deficiency) was performed to identify consensus loci for K-related traits. These consensus MQTL regions are independent of environment × genotype interaction and population type, and hence along-with the novel associations identified, will help in breeding wheat cultivars for high K efficiency.

## Material and Methods

### Plant Material and Phenotyping

A historical bread wheat panel of 150 Pakistani spring wheat varieties acclimated to irrigated, arid, and semi-arid climates was selected for sowing. The panel was categorized into four groups on the basis of release time of cultivars: group-I consisted of 20 varieties of pre-green revolution release (1965 or earlier), group-II consisted of 30 varieties of green revolution release (1965-1979), while group-III and group-IV each had 50 cultivars comprised of post-green revolution release and elite cultivars, respectively (**Table S1**). Seeds of the panel were obtained from National Agricultural Research Centre (NARC), Islamabad, Pakistan and were evaluated for phenotypic purity by five consecutive field trials during 2010-2014 (Ain et al., 2015; Almas et al., 2018).

Cigar roll method proposed by Zhu et al. (2005) was adapted for the hydroponic experiment. Five seeds of each variety were placed on a germination paper of 20 × 20 cm after sterilization and the paper was vertically rolled. Five beakers filled with 200 ml Hoagland solution were prepared, each of which contained 25 cigar rolls for both normal and low K conditions, i.e., 235 ppm-K and 117.5 ppm-K, respectively (Hoagland and Arnon, 1950). Experiment was carried out in three replications for randomization. Plants were kept in a growth chamber having a temperature of 18°C–25°C and 10-hours of light exposure. At 21st day of germination, plants were harvested for further physiological analysis.

Field trials were carried out for three consecutive years from 2016–2018 in triplicated alpha-lattice design for normal K (Control, C) and low K (Treatment, T) conditions, experiments were planted in PVC pipes. Two kg soil (soil to sand ratio of 2:1) was added to each PVC pipe. Random soil samples were tested according to Chen and Ma (2001); sand, silt, and clay were observed at a ratio of 5:2:3, pH of the soil was 7.8, organic matter was 0.70%, and dS/m mean EC value was 3.3. Soil had NPK concentration as 0.73 mg/kg total N, 0.025 mg/kg available P, and 0.06 mg/kg available K. Potash was applied as a source of K, DAP as a source of P, and Urea as a source of N. Plants under normal K conditions (C) were treated with 100% K (0.18 g/kg of soil) while the plants under low K conditions (T) with 50% K (0.09 g/kg of soil). Prior to sowing, seeds were surface sterilized with 25% H_2_O_2_ for 5–10 min followed by washing with distilled water before placing in petri dishes for germination. Once germinated, three seeds were transplanted to field in PVC pipes of 2-feet height and 5-inch diameter at the experimental field of Plant Sciences department, Quaid-i-Azam University, Islamabad, Pakistan (33.7476° N, 73.1381° E).

Agronomic traits such as fresh weight (FW), shoot length (SL), relative water content (RWC), and leaf area (LA) for hydroponic experiment (__H_) while days to maturity (DM), leaf area (LA), plant height (PH), tiller number (TN), spikelet per spike (SpS), grain yield (GY), thousand kernel weight (TKW), biological yield (BY), and harvest index (HI) for field experiment (__F_) were measured according to Pask et al. (2012) and Ain et al. (2015). Chlorophyll contents were measured in cigar trials at 14 days (Chl__14DAG_) and 21 days (Chl__21DAG_) after germination and at heading stage in field trials. Chlorophyll meter, SPAD-502 Konica Minolta sensing Inc., was used to measure the chlorophyll contents. Scanned root images were used to estimate various root parameters (root length, root width, and network area) using GiaRoots software.

### Potassium Estimation

Accuracy evaluation of analytical methods was performed using Duck weed BCR^®^ (BCR-670) certified reference material (Sigma-Aldrich). Oven dried leaf and grain samples (0.5 g) and certified reference material were digested in separate 50 ml conical flasks by adding 7.5 ml (65%) HNO_3_ and 2.5 ml (36%) HCl to each sample. The mixtures were subjected to about 270°C by placing the flasks on a hot plate, the mixtures started evaporating as dense yellowish fumes. Once the yellowness of fumes started to disappear, flasks containing mixtures were removed from the hot plate. Distilled water was added to each flask to raise the final volume to 25 ml. K concentration in leaf samples while zinc (Zn) and iron (Fe) concentration in grain samples of both treatments and certified reference material were all analyzed using atomic absorption spectrometer model WFX-210 (Beijing Beifen-Ruili Analytical Instrument Co., Ltd. China). No significant variation was observed between the atomic absorption results of Duck weed BCR^®^ (BCR-670) certified reference material, i.e., 0.90 g/kg, 20 mg/kg, and 5.39 mg/kg for Fe, Zn, and K, respectively, and the certified values, i.e., 0.94 g/kg, 24 mg/kg, and 5.79 mg/kg for Fe, Zn, and K, respectively. K utilization efficiency (KUtE) was calculated as a ratio between biological yield (grain yield in case of field trial) and K uptake. K uptake efficiency (KUpE) was measured as a ratio between K uptake and K available. Finally, the K use efficiency (KUE) was estimated as a product of KUtE and KUpE (Sandaña, 2016).

### Genotyping

DNA was extracted from 25 days old seedlings following CIMMYT manual (Dreisigacker et al., 2012) and genotyped using 90 K Infinium iSelect SNP array (Ain et al., 2015; Almas et al., 2018). PowerMarker 3.0 was used for estimating genetic similarities among wheat lines with a Dice coefficient based on shared alleles proportion (Liu et al., 2005). Polymorphism information content (PIC) was used to determine genetic diversity at each locus. From 81,587 markers of the genotyped data, markers having mediocre quality in sense of containing indels (sequencing errors) or low raw base quality score (19,810), monomorphic markers (36,765), and markers with less than 5% MAF (4,159) were removed from the genotype dataset before analysis, and finally 20,853 markers were used for association analysis. A physical map of all 21 wheat chromosomes was constructed using the 20,853 polymorphic markers projected onto the newly released wheat reference map (IWGSC *RefSeq v.1.1*), resulting in an average of 795 SNP markers per chromosome. Marker density was highest for B sub-genome and relatively lower for the D sub-genome. SNP dataset can be requested *via* email for further research purposes.

### Statistical Analysis

#### Phenotypes

Descriptive statistics, correlation among traits, and ANOVA were estimated for all the traits using IBM SPSS Statistics 22. Graphical representation of correlation among traits of interest along with histograms and scatterplots was performed in an R package for data visualization, GGally (an extension to ggplot2), in R 3.6.1.

#### Population Structure, Linkage Disequilibrium (LD), and Kinship Matrix

Population structure was analyzed using STRUTURE software, while kinship analysis was carried out in TASSEL 5 software. LD among the markers was estimated for the diversity panel in TASSEL 5 using the observed vs. expected allele frequencies. The *r^2^* value was estimated for pairwise SNPs in a two Mb distance and then averaged across the genome. The LD decay was measured as the distance at which the average *r^2^* between pairwise SNPs dropped to half of its maximum value (Huang et al., 2010). The detailed description of population structure, polymorphism information content (PIC), and minor allele frequencies (MAF) for SNPs was provided in two of our earlier reports of the diversity panel (Ain et al., 2015; Almas et al., 2018).

#### Genome-Wide Association Study

GWAS was conducted by both SL-GWAS (MLM) model and the ML-GWAS models for all the agronomic traits assessed in hydroponic and field trials. The MLM model proposed by Yu and Buckler (2006) was applied in TASSEL 5 to measure the association between phenotype and genotype. Population structure and kinship matrix, that had already been estimated, were used in MLM. Four ML-GWAS models including mrMLM (Wang et al., 2016), FASTmrMLM (Zhang and Tamba, 2018), FASTmrEMMA (Wen et al., 2018), and pLARmEB (Zhang et al., 2017) were applied to the data using an R package mrMLM (https://cran.r-project.org/web/packages/mrMLM/index.html) for ML-GWAS analysis. Critical threshold of significance was set at *P* ≤ 0.000001 (Bonferroni significance threshold = P ≤ 0.000047) for SL-GWAS and LOD ≥ 5 form ML-GWAS, normal significant threshold of ML-GWAS model is LOD ≥ 3 since no Bonferroni is applied. Significance of threshold was kept stringent to enhance the precision of candidate genes identification (Lü et al., 2018). The loci identified by GWAS were further validated for precision, accuracy, and novelty by MQTL analysis. MTA loci were named according to the nomenclature proposed by McCouch (1997). Genomic data visualization was performed in a 2D track plot R package RCircos (https://cran.r-project.org/web/packages/RCircos/index.html).

#### MQTL Characterization

Meta-analysis was performed for four independent studies that had reported QTL for agronomic traits in wheat under K stress (Guo et al., 2012; Kong et al., 2013; Zhao et al., 2014; Gong et al., 2015). Guo et al. (2012) identified 655 QTL linked to nutrient efficiency (N, P, and K) and agronomic traits in recombinant inbred lines (RILs) from a cross Chuan 35050 × Shannong 483. Kong et al. (2013) used 131 RILs derived from a bi-parental cross Chuan 35050 × Shannong 483 to map 167 QTL across hydroponic, pot, and field environments. Zhao et al. (2014) used 168 double haploid lines from a cross Huapei 3 × Yumai 57 to detect 65 QTL across all chromosomes excluding 2B, 5A, and 7B. Gong et al. (2015) used the set of 131 RILs from a cross Chuan 35050 × Shannong 483 to map 127 QTL across 20 chromosomes, except 4D. The detailed description of QTL mapping populations is given in **Table 1**. Relatively high frequency QTL or QTL clusters (130) identified in these four studies were projected to reference genetic map WCGM2017 (Quraishi et al., 2017), having at least 75% common markers with the maps used for QTL detection, before performing MQTL analysis using BioMercator software (Goffinet and Gerber, 2000). Although these four studies evaluated different populations in various hydroponic/pot/field experiments and environmental conditions, their integration provided us a comprehensive view of QTL identified in wheat grown under K stress conditions. QTL clusters belonging to at least two different populations were considered as MQTL, as described by Goffinet and Gerber (2000). The WCGM2017 data is accessible to the scientific community through a web platform allowing to navigate between the genetic regions up to the synteny with grass relatives and ultimately candidate genes. The public web interface named PlantSyntenyViewer available at http://urgi.versailles.inra.fr/synteny-wheat can be used to identify the genetic (markers, QTL, MQTL) and associated genomic (wheat syntenome and syntenic genes from related grasses) data for a translational research approach.

**Table 1:** The description of various QTL studies (related to K-deficiency in bread wheat), used for QTL meta-analysis.

Map	Parent 1	Parent 2	Population	Markers	Marker Type	QTL
Guo et al. (2012)	Chuan35050	Shannong483	RIL	719	Dart, SSR, EST-SSR	655
Kong et al. (2013)	Chuan35050	Shannong483	RIL	719	Dart, SSR, EST-SSR	167
Zhao et al. (2014)	Huapei 3	Yumai 57	DH	323	SSR, EST, iSSR, HMW-GS	65
Gong et al. (2015)	Chuan35050	Shannong483	RIL	719	Dart, SSR, EST-SSR	127

#### Candidate Gene Mining

Genes associated with stable loci from GWAS were predicted on the basis of LD using the EnsemblPlants database available at http://plants.ensembl.org/Triticum_aestivum/Info/Index and the International Wheat Genome Sequencing Consortium (IWGSC) *RefSeq v1.1* annotations (Appels et al., 2018), available at https://wheat-urgi.versailles.inra.fr/Seq-Repository/Annotations. Nearby genes in the linkage regions of stable SNP-trait associations with putative functions that could be related to the trait were selected as candidates.

## Results

### Phenotypic Analysis of Agronomic Traits

**Figure 1:**
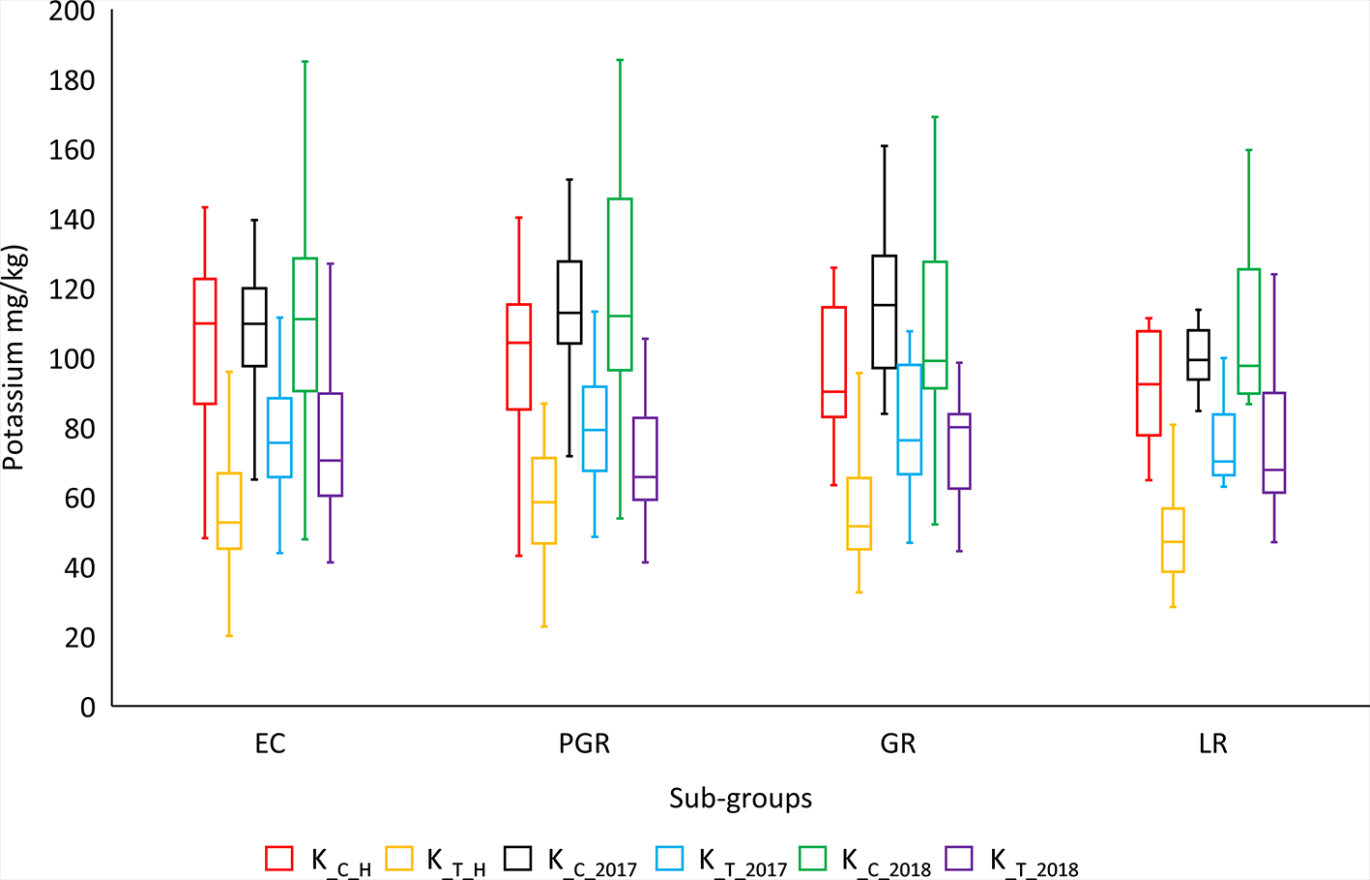
Genotypic distribution of potassium-uptake in historical bread wheat panel of Pakistan. Historical panel consists of four groups of genotypes: EC (elite cultivars), PGR (post green revolution era cultivars), GR (green revolution era cultivars), and LR (landraces). *cf. Trait nomenclature is presented in **Table 2** legends.

ANOVA showed significant variation between genotypes within a treatment and between different treatments for most of the agronomic traits. ANOVA results for important phenotypic and physiological traits along with descriptive statistics are presented in **Table 2**. SpS, TN, GpS, GY, BY, FW, RL, and K-related traits were observed to vary significantly (P < 0.001***) between different treatments across field and hydroponic environments. The four groups of genotypes (landraces, green revolution era cultivars, post-green revolution era cultivars, and elite cultivars) showed a variation in K uptake. Elite cultivars and post green revolution era cultivars generally had a higher K-uptake, green revolution era cultivars had a moderate K-uptake, while landraces had the lowest K-uptake in most of the treatments. An elite cultivar Punjab-11 had a consistently higher K-uptake across all environments on an average basis, i.e., 142.7625 (mg/kg), 95.9675 (mg/kg), 171.22 (mg/kg), 186.6 (mg/kg), 198.1214 (mg/kg), and 147.3462 (mg/kg) in K__C_H_, K__T_H_, K__C_2017_, K__T_2017_, K__C_2018_, and K__T_2018_, respectively (**Figure 1**). These results provide indications of selection for complex traits during the process of breeding improvement. However, to ensure that the sub-populations are not structured in a biased manner for target traits, we performed Shapiro-Wilk and Jarque-Bera normality tests which indicated a normal distribution of target traits across the entire population (**Table S2**).

**Table 2:** Descriptive statistics and analysis of variance (ANOVA) between genotype groups within a treatment and between the treatments.

Trait^a^			GR	FW	RWC	SL	SW	LA__H_	RL__H_	RW	CHL__14DAG_		CHL__21DAG_	Na^+^__H_	K__H_	KUpE__H_	KUtE__H_	KUE__H_
Control	Hydroponics	Mean	94.1	0.10	75.77	19.5	0.3	4.4	7.1	4.7	23.4	23.3		39.1	100.3	0.42	1.1	0.44
		Gen**^†^**	**	**	***	***	**	**	***	**	*		**	***	***	**	**	***
Treatment		Mean	80.4	0.08	73.06	16.2	0.2	3.1	11.3	3.6	22.7	22.2		55.7	30.6	0.47	1.55	0.67
		Gen**^†^**	***	**	**	***	*	**	***	**	*		**	***	***	***	***	***
Genotype × Treatment			***	***	*	***	***	***	***	***	*		**	***	***	**	***	***
**Trait^b^**			**DM**	**CHL**	**PH**	**LA__F_**	**RL__F_**	**SpS**	**GpS**	**TN**	**BY**	**GY**	**TKW**	**Na^+^__F_**	**K__F_**	**KUpE__F_**	**KUtE__F_**	**KUE__F_**
Control	2018	Mean	138	47.4	70.20	15.3	61.3	17.6		2.9	6.8	2.7	37.3	72.6	114.9	0.64	0.02	0.014
		Gen**^†^**	*	*	*	**	***	**		***	***	***	***	***	***	***	**	**
Treatment		Mean	141	43	66.3	12.5	45.6	15.7		1.8	4.6	1.7	35.6	72.2	73.2	0.81	0.02	0.019
		Gen**^†^**	*	-	**	**	***	**		***	***	***	**	***	***	***	***	**
Genotype × Treatment			**	***	**	***	***	***		***	***	***	**	***	***	***	-	***
Control	2017	Mean	135	43.1	70.9	24.6	56.9	17.9	40.7	2	8.8	3.7	44.6	39.1	112.3	0.62	0.03	0.02
		Gen**^†^**	**	*	*	**	**	**	***	*	**	**	***	**	***	***	**	***
Treatment		Mean	140	43	58.7	15	48.5	15.9	28.2	1.3	3.6	1.6	37	30.6	79.8	0.89	0.02	0.018
		Gen**^†^**	**	-	**	**	**	**	***	**	**	*	**	***	***	***	**	***
Genotype × Treatment			***	-	***	***	***	***	***	***	***	***	***	**	***	***	***	*
Control	2016	Mean		41.2	48.6	9.3			46	3.3	6.9	4.8	33.3					
		Gen**^†^**		**	**	**			***	*	**	**	***					
Treatment		Mean		41.3	53.6	6			40.8	2.9	5.7	3.4	30.4					
		Gen**^†^**		**	**	**			**	**	***	*	***					
Genotype × Treatment				-	***	***			***	**	***	***	**					

^a^Germination Rate (GR), Fresh Weight (FW), Relative Water Content (RWC), Shoot Length (SL), Shoot Width (SW), Leaf Area Hydroponics (LA__H_), Root Length Hydroponics (RL__H_), Root Width (RW), Chlorophyll Measured 14 Days After Germination (Chl__14DAG_), Chlorophyll Measured 21 Days After Germination (Chl__21DAG_), Sodium in Leaf Hydroponics (Na_+__H), Potassium in Leaf Hydroponics (K__H_), Potassium Uptake Efficiency Hydroponics (KUpE__H_), Potassium Utilization Efficiency Hydroponics (KUtE__H_), Potassium Use Efficiency Hydroponics (KUE__H_).

^b^Days to Maturity (DM), Chlorophyll at Heading (CHL), Plant Height (PH), Leaf Area Field (LA__F_), Root Length Field (RL__F_), Spikelet Per Spike (SpS), Grains Per Spike (GpS), Tiller Number (TN), Biological Yield (BY), Grain Yield (GY), Thousand Kernel Weight (TKW), Sodium in Leaf Field (Na_+__F), Potassium in Leaf Field (K__F_), Potassium Uptake Efficiency Field (KUpE__F_), Potassium Utilization Efficiency Field (KUtE__F_), Potassium Use Efficiency Field (KUE__F_).

**^†^**ANOVA between genotype groups, based on release year of cultivars (c.f. **Table S1**). Asterisks indicate significance level of variance (* ~ P < 0.05, ** ~ P < 0.01, *** ~ P < 0.001).

**Figure 2:**
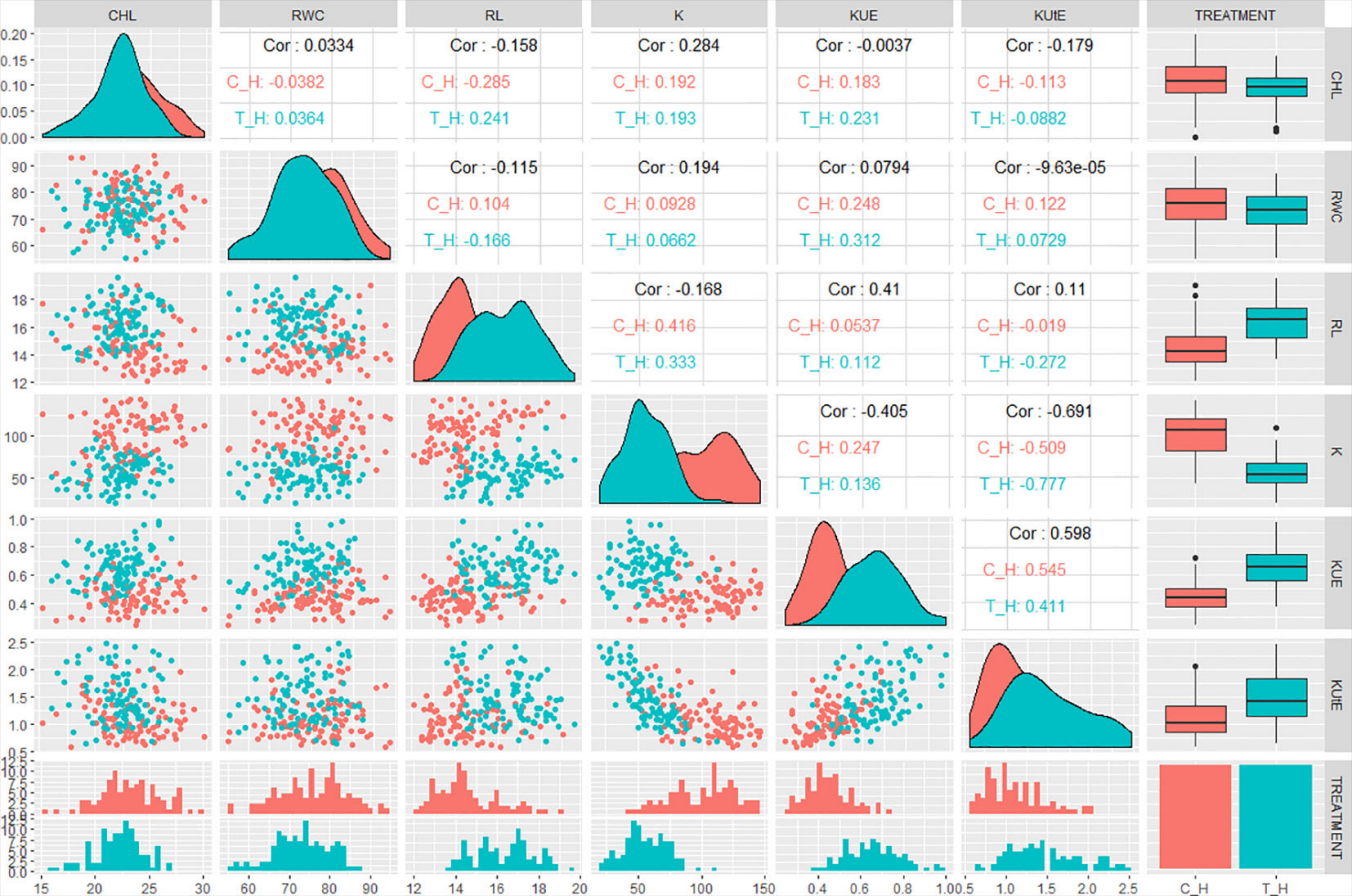
QQ scatterplots, histograms, coefficient of correlation, and box plots between K-use efficiency (KUE) traits from hydroponic experiment. Lower half of matrix and the center line cutting the figure into two triangles represent histograms for each trait. Box plots are presented at the extreme right of upper triangle. Between the histograms in lower triangle are QQ scatter plots. Between the box plots and histograms in the upper triangle are coefficient of correlation (*r* ^2^ values) in control and treatment; *x* and *y* axes of histograms and scatter plots represent phenotypic distribution of traits. *cf. Trait nomenclature is presented in **Table 2** legends.

Correlation analysis indicated significant relationships between the traits of interest. In hydroponic experiment, KUE was positively correlated with CHL, RWC, K, and KUtE in control (0.18, 0.24, 0.24, and 0.54, respectively). In stress condition, KUE had a significant positive correlation with CHL, RWC, RL, K, and KUtE (0.23, 0.31, 0.12, 0.13, and 0.41, respectively). KUtE showed a negative correlation with RL under stress (-0.27), while a highly negative correlation with K in both control and stress treatment (-0.51 and -0.77, respectively). K showed a significant positive correlation with RL in both treatments (0.41 in control, 0.33 in stress), while other relationships were either nonsignificant or slightly significant. Correlation analysis for all the agronomic traits in hydroponic experiment are presented in **Figure 2** and **Table S3**.

**Figure 3:**
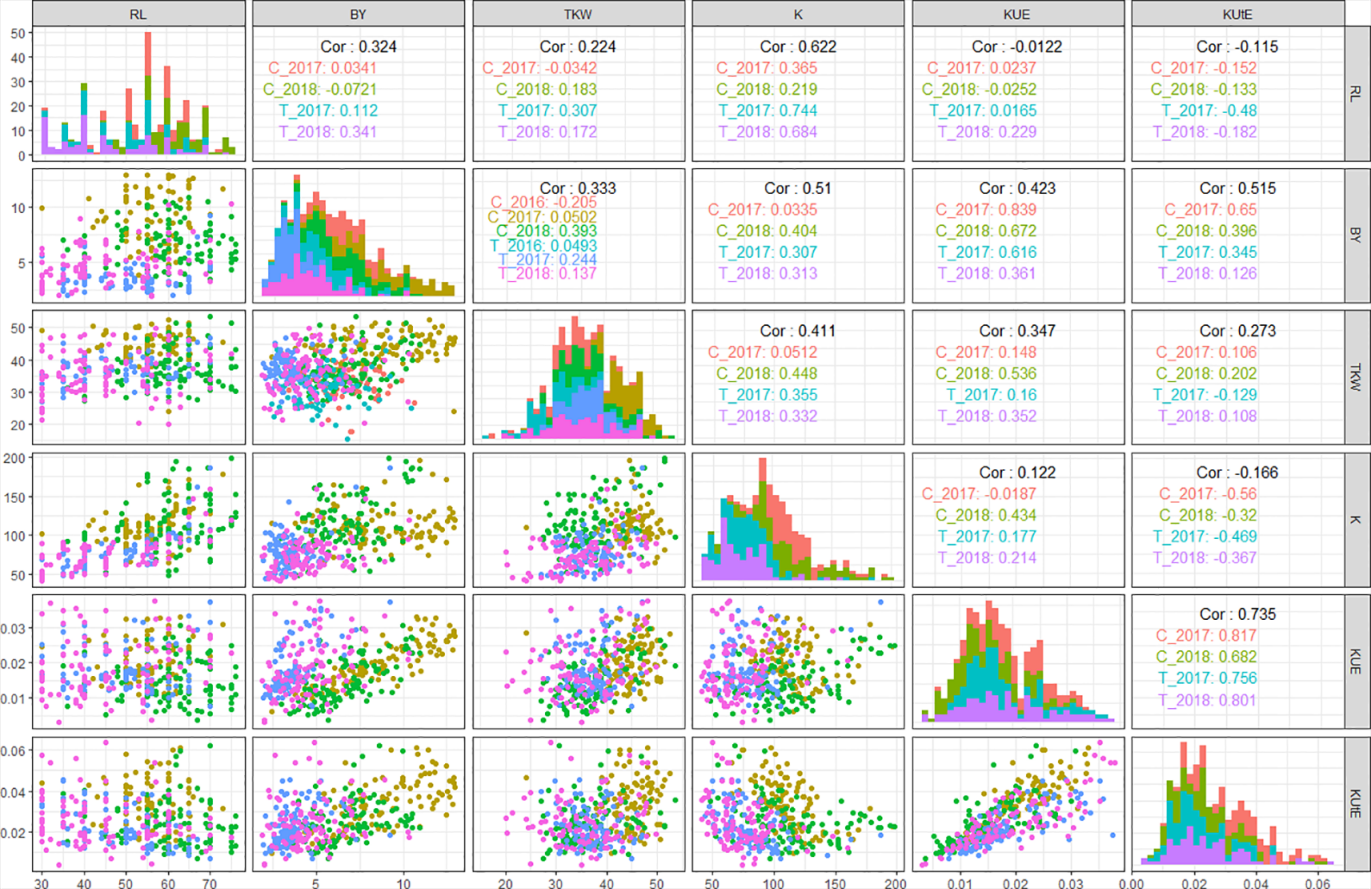
QQ scatterplots, histograms, and coefficient of correlation between K-use efficiency (KUE) traits from field experiments. The plots in center cutting the figure into upper and lower triangles represent histograms. Lower triangle represents QQ scatter plots and upper triangle represents coefficient of correlations (*r* ^2^ values) in different treatments in all field environments; *x* and *y* axes of histograms and scatter plots represent phenotypic distribution of traits. *cf. Trait nomenclature is presented in **Table 2** legends.

In field experiments, KUE positively correlated with BY across all treatments in years 2017 and 2018 (0.83 in C__2017_, 0.61 in T__2017_, 0.67 in C__2018_, and 0.36 in T__2018_). Similarly, KUE showed a highly positive correlation with TKW under both control and stress conditions in 2018 (0.53 in C__2018_ and 0.35 in T__2018_) while a less significant positive correlation with TKW in 2017 (0.14 in C__2017_ and 0.16 in T__2017_). KUtE had an extremely positive correlation with KUE across all treatments (0.81 in C__2017_, 0.75 in T__2017_, 0.68 in C__2018_, and 0.8 in T__2018_) while a completely reversed relationship with K under both treatments in 2017 and 2018 experimental seasons (-0.56 in C__2017_, -0.46 in T__2017_, -0.32 in C__2018_, and -0.36 in T__2018_). K showed significant positive correlation with RL in C__2017_ (0.36), T__2017_ (0.74), C__2018_ (0.21), and T__2018_ (0.68). Similarly, K and BY were positively correlated in T__2017_, C__2018_, and T__2018_ with the *r* values of 0.3, 0.4, and 0.31, respectively. TKW and K showed a highly positive correlation in T__2017_ (0.35), C__2018_ (0.44), and T__2018_ (0.33). The detailed analysis of correlation among all the agronomic and physiological traits across all the field environments is presented in **Figure 3** and **Table S4**.

### SNP Marker Analysis, Population Structure, and LD

**Figure 4:**
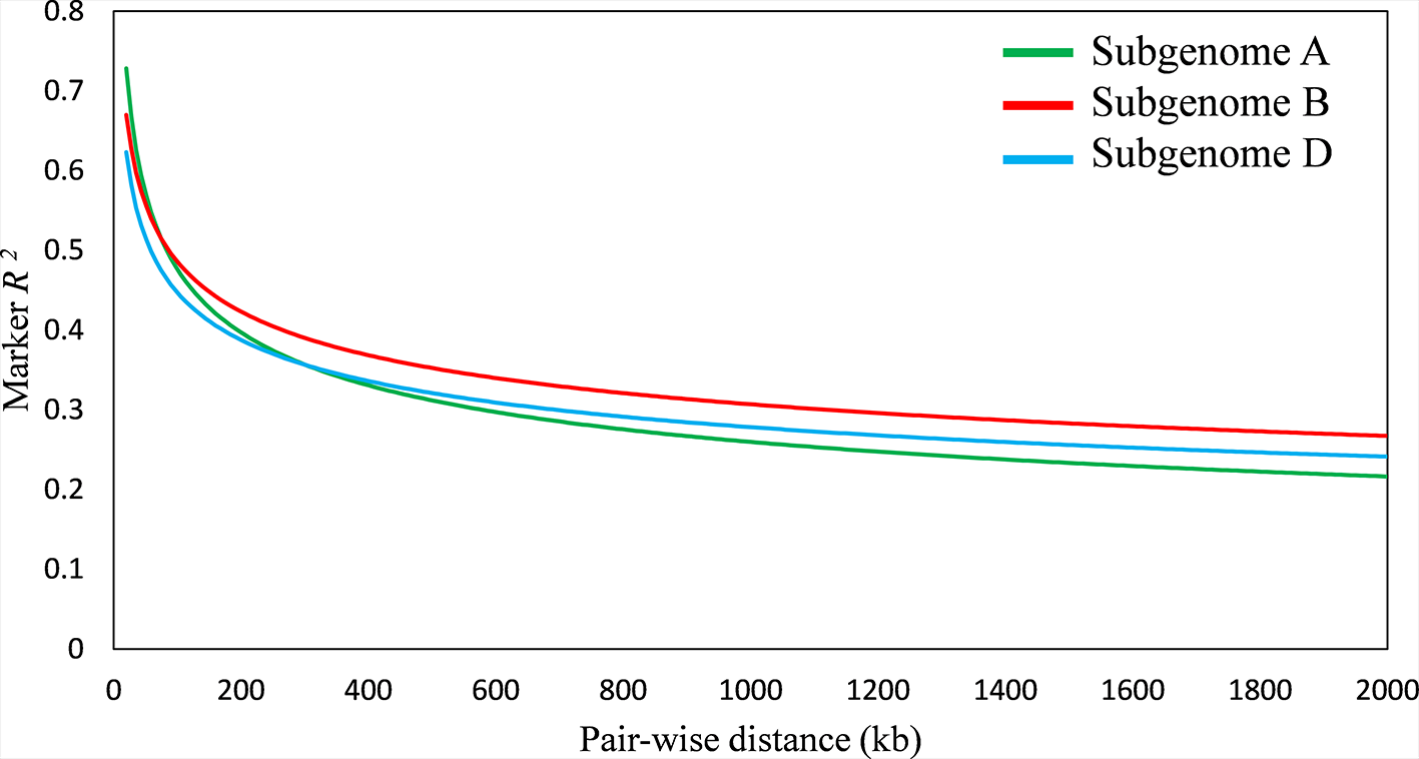
Linkage disequilibrium (LD) decay plot of wheat sub-genome A, B, and D; *x*-axis represents distance between single nucleotide polymorphisms (SNPs) in kb, *y*-axis represents average *r^2^* between pairwise SNPs.

A total of 20,853 SNP markers with MAF ≥ 0.05 was used for GWAS analysis. These markers were obtained after trimming the original set of 81,587 SNPs for missing values, inadequate quality markers (with sequencing errors, indels), and markers with MAF ≤ 0.05. Population structure analysis divided the panel into seven subgroups of cultivars, details were provided in our previous report on the diversity panel (Ain et al., 2015). The extent of LD was estimated for the diversity panel using TASSEL software. It indicated the B sub-genome to have highest LD, followed by D and A sub-genomes, respectively. LD decreased with the increase in physical distance between marker loci. Average LD decay was observed after 300 kb in A sub-genome, after 800 kb in B sub-genome, and after 500 kb in D sub-genome (**Figure 4**).

### MTA and Loci Identification

SL-GWAS method (MLM) identified 661 MTA. Out of these, six MTA with -log10p ≥ 5 were considered as significant and used for further analysis (**Table S5**). ML-GWAS identified 1,319 MTA for all the traits across all four experimental environments. Four ML-GWAS models were used for association analysis; pLARmEB identified highest number of MTA with 567, FASTmrMLM 477, mrMLM 192, and FASTmrEMMA identified 83 MTA. These MTA were screened for significance of threshold at LOD score ≥ 5 which resulted in 528 significant MTA. These 528 MTA included all four GWAS models; pLARmEB identified 279 MTA, FASTmrMLM 213, mrMLM 35, while FASTmrEMMA identified only one MTA with LOD score greater than five (**Table S6**).

**Figure 5:**
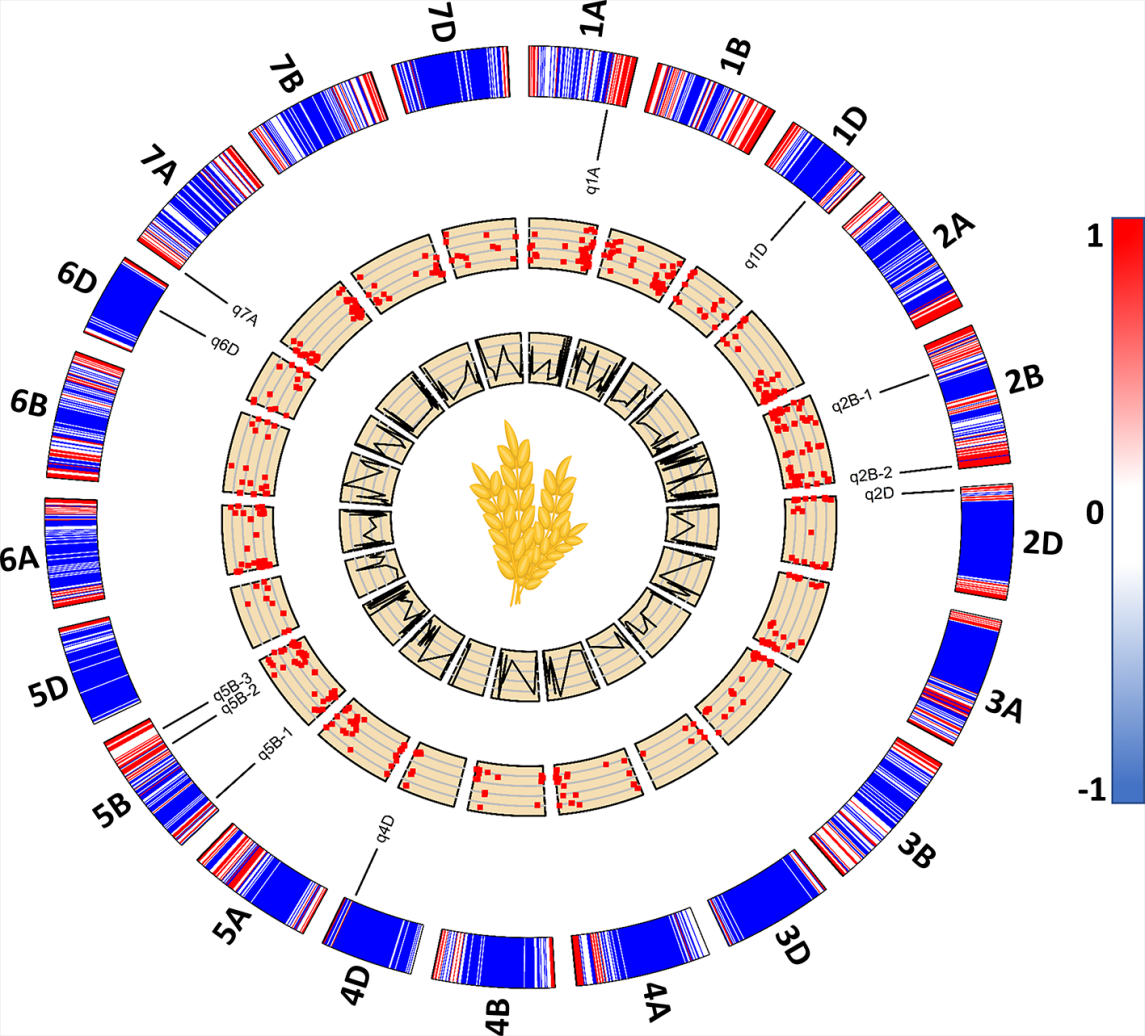
A 2D track plot visualizing genomic data. The outermost track represents heatmap of marker density in the genotype data used for genome-wide association studies (GWAS) and the placement of stable loci on wheat chromosomes with respect to their physical position. Scatterplots represent significant marker-trait associations (MTA) with lower to higher -log10p from inside out. The innermost line plots represent the LOD score threshold of significant MTA.

Among the 534 significant SNPs from both SL-GWAS and ML-GWAS, 54 were detected by at least two different GWAS models (**Table S7**). These 54 SNPs distributed over 52 loci on wheat genome; 21 loci on sub-genome A, 20 on sub-genome B, and 11 on sub-genome D (**Table S8**). These 52 loci were identified on 18 wheat chromosomes, excluding 3D, 4A, and 4B. Among the 52 significant loci, 11 were consistent across more than one experimental environment. These 11 loci, detected by multiple GWAS models and consistent across multiple environments, were considered as stable loci for candidate gene prediction. These stable genomic regions were present on eight wheat chromosomes; 1A, 1D, 2B-I, 2B-II, 2D, 4D, 5B-I, 5B-II, 5B-III, 6D, and 7A (**Figure 5**, **Table 3**). MTA loci were named according to the nomenclature assigned by McCouch (1997) e.g., *q1A* refers to the stable locus on chromosome 1A, *q2B-1* refers to the 1st stable locus on chromosome 2B, while *q2B-2* refers to the 2nd stable locus on chromosome 2B, and so on.

**Table 3:** Stable marker-trait associations (MTA) identified by different genome-wide association studies (GWAS) models.

MTA Loci	SNP ID^†^	Trait^a^	GWAS Model^b^	Chr	Position^c^	-log10p value	LOD score
*q1A*	IWB20856	SpL__C_2018_	pLARmEB			6.77	5.94
		FW__T_H_	**FASTmrMLM**	1A	485002381	12.64	11.67
*q1D*	IWB41036	HI__C_2017_	FASTmrMLM			7.87	7.01
		RL__C_H_	pLARmEB			7.44	6.58
		FW__T_H_	**FASTmrMLM**	1D	336365267	11.88	10.92
		FW__T_H_	pLARmEB			7.39	6.54
*q2B-1*	IWB4614	KUE__T_2017_	**pLARmEB**	2B	180616389	7.69	6.83
		SpL__C_2018_	FASTmrMLM			5.80	5.01
		SpL__C_2018_	pLARmEB			6.01	5.20
*q2B-2*	IWB7106	KUtE__C_2017_	**pLARmEB**	2B	783226928	7.08	6.24
		CHL__T_2016_	MLM			5.11	
*q2D*	IWB740	KUE__C_2017_	**FASTmrMLM**	2D	14395525	14.38	13.39
		KUE__T_2018_	FASTmrMLM			6.25	5.44
		KUE__T_2018_	pLARmEB			6.54	5.72
*q4D*	IWA410	KUE__T_2017_	pLARmEB			12.60	11.63
		KUE__C_2018_	**FASTmrMLM**	4D	453220983	15.95	14.94
		KUE__C_2018_	pLARmEB			6.44	5.62
		SW__T_H_	FASTmrMLM			11.69	10.74
*q5B-1*	IWB58160	KUtE__T_2017_	**pLARmEB**	5B	64733088	13.19	12.21
		KUE__C_2018_	FASTmrMLM			5.89	5.09
*q5B-2*	IWB38863	KUtE__T_2017_	pLARmEB			9.52	8.61
		KUE__C_2018_	FASTmrMLM			9.33	8.43
		KUE__C_2018_	**pLARmEB**	5B	536515270	15.95	15.06
*q5B-3*	IWB7750	KUtE__C_2017_	pLARmEB			6.06	5.25
		KUE__T_2018_	**FASTmrMLM**	5B	558346572	6.59	5.76
*q6D*	IWB35315	FW__C_H_	**FASTmrMLM**	6D	309588003	6.98	6.15
		Na^+^__C_2018_	pLARmEB			6.06	5.25
*q7A*	IWB33486	DM__C_2018_	mrMLM			7.20	6.35
		DM__C_2018_	FASTmrMLM			7.20	6.36
		DM__C_2018_	pLARmEB			7.20	6.36
		FW__T_H_	**pLARmEB**	7A	1699666	11.01	10.07

^†^Universal IDs of SNP markers from IWGSC RefSeq v1.1.

^a^Trait nomenclature is presented in **Table 2** legends.

^b^Models highlighted in bold detected SNP with highest LOD.

^c^Position of SNP marker from IWGSC RefSeq v1.1.

### MQTL Identification

**Figure 6:**
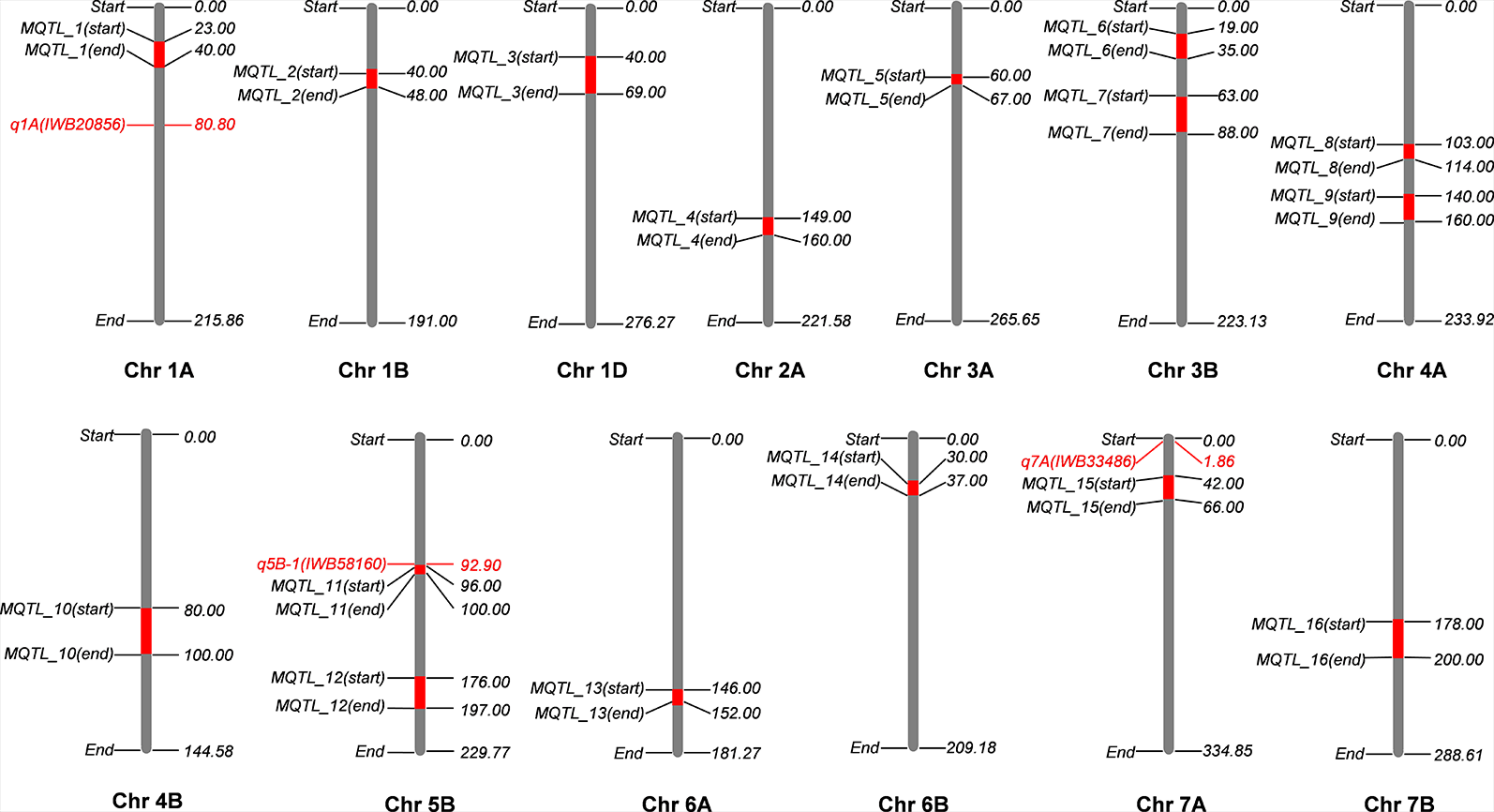
Meta-QTL (MQTL) identified on wheat consensus genetic map 2017 (WCGM2017) using four independent quantitative trait loci (QTL) studies. One stable locus from genome-wide association studies (GWAS) results colocalizes with an MQTL at chromosome 5B (*q5B-1* with *MQTL__11_*), only three detected loci are present on chromosomes harboring MQTL. *cf. Information of QTL studies is presented in materials and methods.

Genetic maps used for the QTL studies (cf. material and methods) were projected on to WCGM2017 consensus map, with at least 75% common markers between any single genetic map of linkage analysis (QTL) and WCGM2017. Before doing the meta-analysis of different populations, meta-analysis of each population to identify consensus regions within the population was also conducted. The QTL cluster belonging to two or more different mapping populations was subjected to meta-analysis for the calculation of MQTL. Overall, 130 QTL (**Table S9**) were used to calculate 16 MQTL on 13 wheat chromosomes; 1A, 1B, 1D, 2A, 3A, 3B-I, 3B-II, 4A-I, 4A-II, 4B, 5B-I, 5B-II, 6A, 6B, 7A, and 7B (**Figure 6**, **Table 4**). These 16 MQTL regions, independent of genotype × environment interaction, are of immense genetic importance considering their stability across multiple populations and environments. One out of the 11 SNP loci (i.e., *q5B-1*) from GWAS resided close to *MQTL__11_* present on chromosome 5B. This SNP was detected by two GWAS models (pLARmEB and FASTmrMLM) with a LOD score of 12.2 and -log10p 13.19 and associated with two K related traits in two environments (KUtE__T_2017_, KUE__C_2018_). All other 10 MTA were novel associations.

**Table 4:** Meta-QTL (MQTL) identified on WCGM for Potassium related traits.

MQTL	Map Name	QTL	Chr	Position^a^
MQTL__1_	Kong et al. (2013); Gong et al. (2015)	13	1A	23-40
MQTL__2_	Guo et al. (2012); Gong et al. (2015)	8	1B	40-48
MQTL__3_	Kong et al. (2013); Zhao et al. (2014); Gong et al. (2015)	5	1D	40-69
MQTL__4_	Zhao et al. (2014); Gong et al. (2015)	5	2A	149-160
MQTL__5_	Kong et al. (2013); Gong et al. (2015)	6	3A	60-67
MQTL__6_	Zhao et al. (2014); Gong et al. (2015)	4	3B	19-35
MQTL__7_	Zhao et al. (2014); Gong et al. (2015)	5	3B	63-88
MQTL__8_	Guo et al. (2012); Gong et al. (2015)	2	4A	103-114
MQTL__9_	Zhao et al. (2014); Gong et al. (2015)	6	4A	140-160
MQTL__10_	Guo et al. (2012); Zhao et al. (2014); Gong et al. (2015)	6	4B	80-100
MQTL__11_	Zhao et al. (2014); Gong et al. (2015)	3	5B	96-100
MQTL__12_	Zhao et al. (2014); Gong et al. (2015)	2	5B	176-197
MQTL__13_	Kong et al. (2013); Gong et al. (2015)	4	6A	146-152
MQTL__14_	Kong et al. (2013); Gong et al. (2015)	3	6B	30-37
MQTL__15_	Guo et al. (2012); Gong et al. (2015)	3	7A	42-66
MQTL__16_	Zhao et al. (2014); Gong et al. (2015)	3	7B	178-200

^a^QTL position (cM) on WCGM 2017.

### Candidate Gene Prediction and Annotation

Putative genes related to traits in the associated genomic regions of 11 stable MTA loci were selected as candidates that resulted in 20 genes on the wheat reference genome assembly IWGSC *RefSeq v1.1* (**Table 5**). Among these, eighteen genes were annotated for functional protein involved in key cellular and biological pathways in wheat and its grass relatives, two genes resulted in hypothetical proteins. Annotated proteins included essential proteins for plant growth and development, and sustainability under abiotic stress environments: *CCAAT-binding transcription factor A, Cytochrome P450 superfamily, COBRA-like protein, GLU1B, RING-type E3 ubiquitin transferase, Arginase, Bidirectional sugar transporter SWEET, Calmodulin-binding family protein, Serine/threonine kinase, UTP–glucose-1-phosphate uridylyltransferase, Photosystem II reaction center protein L, Auxin-responsive protein,* and *Acyl-transferase.*

**Table 5:** Annotation of candidate genes associated to stable single nucleotide polymorphism (SNP) variants.

Gene	Var^a^	Chr	Start	End	SNP ID^b^	LOD Score	Annotation^c^
*TraesCS1A02G288500*	4	1A	485359631	485363435	IWB20856	11.67	*CCAAT-binding transcription factor A*
*TraesCS1D02G245600*	1	1D	337217074	337219376	IWB41036	10.92	*Cytochrome P450 superfamily*
*TraesCS2B02G201400*	1	2B	180558389	180560528	IWB4614	6.83	*COBRA-like protein*
*TraesCS2B02G201500*	1	2B	180568362	180572362	IWB4614	6.83	*COBRA-like protein*
*TraesCS2B02G599800*	1	2B	782533511	782538118	IWB7106	6.24	*GLU1B*
*TraesCS2B02G601300*	1	2B	784268263	784269876	IWB7106	6.24	*RING-type E3 ubiquitin transferase*
*TraesCS2D02G034900*	2	2D	13439965	13444862	IWB740	13.38	*Arginase*
*TraesCS2D02G042400*	1	2D	15193845	15197322	IWB740	13.38	*Bidirectional sugar transporter SWEET*
*TraesCS2D02G042500*	1	2D	15228745	15230408	IWB740	13.38	*Bidirectional sugar transporter SWEET*
*TraesCS2D02G042600*	1	2D	15264456	15266348	IWB740	13.38	*Bidirectional sugar transporter SWEET*
*TraesCS4D02G281600*	1	4D	452795390	452798654	IWA410	14.94	*Calmodulin-binding family protein (At)*
*TraesCS5B02G059000*	2	5B	64731218	64744679	IWB58160	12.21	*Serine/threonine kinase*
*TraesCS5B02G356300*	2	5B	536045679	536052111	IWB38863	15.06	*UTP–glucose-1-phosphate uridylyltransferase*
*TraesCS5B02G380100*	1	5B	558109705	558109821	IWB7750	5.76	*Photosystem II reaction center protein L*
*TraesCS5B02G381500*	1	5B	559763470	559765063	IWB7750	5.76	*Non-specific serine/threonine protein kinase*
*TraesCS5B02G381800*	1	5B	559778240	559779074	IWB7750	5.76	*Auxin-responsive protein*
*TraesCS5B02G381900*	1	5B	559990916	559992903	IWB7750	5.76	*Auxin-responsive protein*
*TraesCS6D02G219200*	1	6D	309040962	309042473	IWB35315	6.15	metal ion binding
*TraesCS6D02G219600*	1	6D	309949038	309951388	IWB35315	6.15	cellular biosynthesis
*TraesCS7A02G002700*	1	7A	1690697	1692368	IWB33486	10.07	*Acyl-transferase*

^a^Alternate splicing variants (mRNA).

^b^Universal IDs of SNPs associated with candidate genes.

^c^Underlined annotations are functional roles of hypothetical proteins.

## Discussion

### Significance of ML-GWAS Models

Most of the complex traits like KUE are dominated by major genes, the one-dimensional model can not detect associations with the variation of polygenes due to the limitations of the model (Lü et al., 2018). Several shortcomings of the single-locus models have been discussed in the recent years, e.g., the general linear models do not use kinship as co-variates (Pace et al., 2015), which results in the high proportion of false positives. The problem with the mixed linear models is that they use a highly stringent criteria of Bonferroni correction that, often results in the loss of many significant SNPs (Gordon et al., 2016). Multi-locus models help ramifying these restrictions caused by single-locus models. In present study, highly significant SNPs identified by different models were: FASTmrEMMA = 1, MLM = 6, mrMLM = 35, FASTmrMLM = 213, and pLARmEB = 279 (**Tables S5** and **S6**). These results indicate and further validate the argument that ML-GWAS models are comparatively better to study the effects of maximum genetic variants in a population. Several studies have individually illustrated the quality and effectiveness of all of these ML-GWAS models (Wang et al., 2016; Zhang et al., 2017; Wen et al., 2018; Zhang and Tamba, 2018).

### Variation Among Different ML-GWAS Models

Four ML-GWAS models detected 1319 significant SNPs with a logarithm of odds three or higher that were later screened for a more stringent criterion for gene prediction. However, among these four models, pLARmEB detected the greatest number of MTA with 567 MTA that had LOD score ≥ 3 (279 with LOD ≥ 5), FASTmrMLM identified 477 MTA with LOD score ≥ 3 (213 with LOD ≥ 5), mrMLM detected 192 MTA with LOD score ≥ 3 (35 with LOD ≥ 5), while FASTmrEMMA detected the lowest number of 83 MTA with LOD score ≥ 3 (1 MTA with LOD ≥ 5). The highest maximum LOD score was observed in pLARmEB (15.19), while the lowest maximum was detected in FASTmrEMMA (5.3). All methods except FASTmrEMMA had a maximum LOD score of greater than 10 for at least one of the associated SNPs. All these findings were consistent with the study of Lü et al. (2018) who used ML-GWAS models to dissect photosynthesis-related traits in soybean. The maximum *r* ^2^ was observed in FASTmrMLM (60.18%), followed by pLARmEB (57%), mrMLM (56.16%), and FASTmrEMMA (17.62%). Overall, these results suggest the dominance of pLARmEB over the other models used in this study. However, the minimum *r* ^2^ observed in mrMLM was *r* ^2^ = 3.9, while the minimum value for all other models was less than 0.01, which indicates that mrMLM can detect major and effective SNPs as compared to other models.

### Novel SNP-Trait Associations and MQTL

The ultimate goal of all the breeding programs is to achieve a high grain yield in moderate to stress environments (Pont et al., 2013; Ain et al., 2015). Therefore, the identification of major loci associated to yield components, for instance KUE in K-stress environment, and their integration with meta-analysis provides an extremely useful breeding approach. Here, 11 loci from GWAS and 16 MQTL from meta-analysis were cross checked to see if the associations from this study were close to the MQTL regions. Only one locus *q5B-1* (IWB38863) was detected in a close proximity to *MQTL__11_* while the other 10 loci were novel associations, eight loci were identified on different chromosome from MQTL. These 11 loci from GWAS were critically screened to reduce the possibility of false positives and are thus presumed as true genetic variants. MQTL are also an important target for breeding potential as they reduce the confidence interval by integrating different independent analyses in one place. Narrowing down a QTL confidence interval is a key step towards a precise search of candidate genes.

The 11 identified loci were associated with important agronomic traits such as biological yield, root architecture, chlorophyll, and potassium use efficiency (**Table 3**). Seven loci including *q2B-1*, *q2B-2*, *q2D*, *q4D*, *q5B-1*, *q5B-2*, and *q5B-3* associated to KUE and KUtE along with other agronomic traits. This provides a compelling evidence that KUE and KUtE have a major influence on the grain yield. Plants invest a greater proportion of photosynthates in the roots and altering the root morphology to enhance exploration of the soil volume (Hermans et al., 2006; Bucher, 2007; Hammond and White, 2008). IWB41036 (*q1D)* was associated with RL, HI, and FW, which implicates the role of root architecture in KUE and overall plant growth. Another stable locus *q2B-2* (IWB7106) encoded KUtE__C_2017_ and CHL__T_2016_, which indicate that K plays a key role in photosynthesis (D T Clarkson and Hanson, 1980; Pettigrew, 2008; Wang and Wu, 2017). Ion channels regulate osmotic balance during environmental stress (Haynes, 1990), SNP IWB35315 at *q6D* was linked to FW__C_H_ and Na^+^__C_2018_. Moreover, enhancing K acquisition improves salinity tolerance of plants by Na^+^ exclusion (Chakraborty et al., 2016). Five of the 11 stable loci had SNPs from hydroponics as well as field traits (**Table 3**), which suggests that the hydroponic experiment for K-deficiency stress can be used to evaluate a large-scale field study.

### Genes–Putative Candidates

Plants respond to environmental stress such as nutrient deficiencies by a multigene regulation. Putative genes selected as candidates were annotated for some key proteins involved in plant growth, sugar metabolism, nutrient transport, and immunity to abiotic/biotic stress factors. *TraesCS1A02G288500* was found in the region of IWB20856 (*q1A*, LOD 11.67), this gene has 4 splicing variants and was annotated for *CCAAT-binding transcription factor A*. This protein increases grain yield of wheat in low nutrient input cropping systems (Qu et al., 2015). IWB4614 (*q2B-1*) encoded *TraesCS2B02G201400* and *TraesCS2B02G201500* genes responsible for *COBRA-like protein* which has been reported to increase grain yield in maize (Hochholdinger et al., 2008) and grain yield and nutrient uptake in rice (Li et al., 2003). *GLU1* protein is coded by 4 genes in wheat genome: *GLU1A*, *GLU1B*, *GLU1C*, and *GLU1D* (Uniprot: Q1XIR9, Q1XH05, Q1XH04, and D5MTF8). This gene has been characterized in wheat and rye (Sue et al., 2006), its beta-glucosidase activity helps in sugar metabolism and defense against pathogens which makes it an important candidate as a grain yield enhancer during abiotic environmental stress. *GLU1B* was annotated from *TraesCS2B02G599800* present at *q2B-2* (IWB7106). Another gene at this locus annotated *RING-type E3 ubiquitin transferase*, an associated QTL is reported for grain width and weight in rice (Song et al., 2007). SWEET (sugar will eventually be exported transporters) family proteins regulate plant nectar production and seed development, these are also involved in nutrition of pathogens and symbionts that help plants in nutrient uptake (Chen et al., 2010). A major locus *q2D* (IWB740) associated to KUE__C_2017_ and KUE__T_2018_ was found in linkage with 3 genes (*TraesCS2D02G042400*, *TraesCS2D02G042500*, and *TraesCS2D02G042600*) encoding *Bidirectional sugar transporter SWEET*. IWA410 at *q4D* (KUE__T_2017_, KUE__C_2018_, and SW__T_H_) harbored *TraesCS4D02G281600*, *Calmodulin-binding family protein* has been characterized in arabidopsis ortholog of this gene. Calmodulin proteins regulate ion channels such as KCa channel, their expression is enhanced in response to abiotic stresses (Virdi et al., 2015). SNP IWB38863 at *q5B-2* was identified with the highest LOD score among stable loci, its corresponding gene *TraesCS5B02G356300* encoded *UTP–glucose-1-phosphate uridylyltransferase* which is a highly conserved gene among eukaryotes and regulates glucose metabolism and is therefore a key component of cellular respiration and energy production (Roeben et al., 2006). Auxins are involved in a multitude of plant growth regulation pathways and are present in almost all parts of plant including stem, leaf, flower, and roots, they are the first plant hormones to be discovered (Kazan, 2013). *TraesCS5B02G381800* and *TraesCS5B02G381900* at *q5B-3* linked to IWB7750 encoded *Auxin-responsive protein*. *Auxin responsive proteins* have been observed to play a key role in root development to enhance nutrient acquisition under stress (Kiba et al., 2010).

Functional validation of candidate genes identified here, 11 loci, and 16 MQTL are potential breeding targets for future studies. Moreover, these findings suggest that ML-GWAS models can target more true genetic variants as compared to SL-GWAS models and the integration of these models with meta-analysis will lead towards the precise dissection of complex traits.

## Data Availability Statement

The datasets generated for this study can be found in the EVA, Project: PRJEB36127; Analyses: ERZ1283759.

## Ethics Statement

All research has been conducted to satisfy Pakistan Agricultural Research Council Act and Quaid-i-Azam University Pakistan research safety standards.

## Author Contributions

UQ conceived and designed the study. LS, TA, and SLa performed the experiments. LS, MT, MU, and SLa analyzed the data. LS performed the statistical data analysis and visualizations. LS and UQ wrote the original draft. UQ, SLi, LS, and XL reviewed and edited the manuscript. UQ and SLi provided the resources. All authors read and approved the last version.

## Funding

This study was supported by the funding from: 1) Higher Education Commission Pakistan project (HEC NRPU-3825); 2) the Knowledge Innovation Program of Chinese Academy of Agricultural Sciences, Central Public-Interest Scientific Institution Basal Research Fund, the Chinese Agricultural Research System (CARS-13).

## Conflict of Interest

The authors declare that the research was conducted in the absence of any commercial or financial relationships that could be construed as a potential conflict of interest.
